# Microdeletion on chromosome 8p23.1 in a familial form of severe Buruli ulcer

**DOI:** 10.1371/journal.pntd.0006429

**Published:** 2018-04-30

**Authors:** Quentin B. Vincent, Aziz Belkadi, Cindy Fayard, Estelle Marion, Ambroise Adeye, Marie-Françoise Ardant, Christian R. Johnson, Didier Agossadou, Lazaro Lorenzo, Julien Guergnon, Christine Bole-Feysot, Jeremy Manry, Patrick Nitschké, Ioannis Theodorou, Jean-Laurent Casanova, Laurent Marsollier, Annick Chauty, Laurent Abel, Alexandre Alcaïs, Franco-Beninese Buruli Research Group

**Affiliations:** 1 Laboratory of Human Genetics of Infectious Diseases, Necker Branch, Institut National de la Santé et de la Recherche Médicale (INSERM) UMR-1163, Paris, France; 2 Imagine Institute, Paris Descartes—Sorbonne Paris Cité University, Paris, France; 3 Department of Radiology, Kremlin-Bicêtre Hospital, Paris, France; 4 Center for Research in Cancerology & Immunology Nantes-Angers (CRCNA), INSERM, Nantes University, Angers University, Angers, France; 5 Centre de Dépistage et de Traitement de la Lèpre et de l'Ulcère de Buruli (CDTLUB), Fondation Raoul Follereau, Pobe, Benin; 6 Fondation Raoul Follereau, Paris, France; 7 Centre Interfacultaire de Formation et de Recherche en Environnement pour le Développement Durable, Université d’Abomey-Calavi, Cotonou, Benin; 8 Leprosy and Buruli Ulcer national control program, Beninese Ministry of Health, Cotonou, Benin; 9 INSERM UMR S 945, Pierre et Marie Curie University, Paris, France; 10 Genomic Core Facility, Paris Descartes—Sorbonne Paris Cité University, Imagine Institute, INSERM UMR-1163, Paris, France; 11 Bioinformatics Core Facility, Paris Descartes—Sorbonne Paris Cité University, Imagine Institute, INSERM UMR-1163, Paris, France; 12 Center for Immunology and Infectious Diseases, INSERM UMR S 1135, Pierre et Marie Curie University, Paris, France; 13 Department of Immunology, Pitié-Salpêtrière Hospital, Paris, France; 14 St. Giles Laboratory of Human Genetics of Infectious Diseases, Rockefeller Branch, Rockefeller University, New York, United States of America; 15 Howard Hughes Medical Institute, New York, United States of America; 16 Pediatric Hematology-Immunology Unit, Necker Hospital for Sick Children, Paris, France; Kwame Nkrumah University of Science and Technology, GHANA

## Abstract

Buruli ulcer (BU), the third most frequent mycobacteriosis worldwide, is a neglected tropical disease caused by *Mycobacterium ulcerans*. We report the clinical description and extensive genetic analysis of a consanguineous family from Benin comprising two cases of unusually severe non-ulcerative BU. The index case was the most severe of over 2,000 BU cases treated at the Centre de Dépistage et de Traitement de la Lèpre et de l’Ulcère de Buruli, Pobe, Benin, since its opening in 2003. The infection spread to all limbs with PCR-confirmed skin, bone and joint infections. Genome-wide linkage analysis of seven family members was performed and whole-exome sequencing of both patients was obtained. A 37 kilobases homozygous deletion confirmed by targeted resequencing and located within a linkage region on chromosome 8 was identified in both patients but was absent from unaffected siblings. We further assessed the presence of this deletion on genotyping data from 803 independent local individuals (402 BU cases and 401 BU-free controls). Two BU cases were predicted to be homozygous carriers while none was identified in the control group. The deleted region is located close to a cluster of beta-defensin coding genes and contains a long non-coding (linc) RNA gene previously shown to display highest expression values in the skin. This first report of a microdeletion co-segregating with severe BU in a large family supports the view of a key role of human genetics in the natural history of the disease.

## Author summary

Buruli ulcer (BU) is a tropical infectious disease caused by *Mycobacterium ulcerans*. Although being the third most common mycobacterial disease in the world after tuberculosis and leprosy, BU remains a neglected tropical disease and an emerging health emergency in several developing countries. It causes profound skin ulcerations and eventually bone infections. Life-long functional sequelae are observed in more than 20% of patients, most of whom are children. Several observations, in particular the large variability in the clinical severity of the disease after infection, suggested the role of human genetic factors in the development of BU. We report the case of a 5-year old girl from Benin, born of consanguineous parents, who suffered from extensive dissemination of the mycobacterium in the skin, bones and joints. One of her siblings was also affected. The deep genetic exploration of this family led to the identification of a small deletion on chromosome 8 in both patients but absent from unaffected siblings. Interestingly, the deletion is located within a region containing genes encoding for beta-defensins, a family of antimicrobial peptides involved in both innate immunity and healing process of skin wounds. This first report of a microdeletion associated with severe BU in a large family supports the view of a key role of human genetics in the natural history of the disease.

## Introduction

Buruli ulcer (BU), caused by *Mycobacterium ulcerans*, is the third most frequent mycobacteriosis worldwide, after tuberculosis and leprosy [1]. It mostly affects rural areas of tropical countries. No reliable estimate of global incidence is currently available but West Africa, annually reporting several thousand of cases, is considered as the principal endemic zone [2]. However, the incidence of BU is currently declining in several African countries including Benin. In 2016, 1,676 BU patients were reported to the World Health Organization (WHO) by African countries as opposed to 5,029 in 2009 (http://www.who.int/gho/neglected_diseases/buruli_ulcer/en/). The reasons for this decline are presently unknown although a role of the introduction of control strategies has been suggested [3].

BU is a devastating necrotizing skin infection classically characterized by pre-ulcerative lesions (nodules, plaques, edematous infiltration), eventually developing into deep ulcers with undermined edges. BU causes life-long functional sequelae in more than 20% of patients, most of whom are children [4]. The occurrence of sequelae in BU patients is not evenly distributed, and severe, sequelae-prone, BU forms have been defined as presentation with edema, osteomyelitis, or large (≥15 cm in diameter) or multifocal lesions [4, 5]. The unexplained variability in the clinical presentation of BU [4–6] together with the indication of familial clustering of cases [7, 8] suggest a role of host genetic factors in the natural history of BU in humans.

This hypothesis is consistent with the discovery of Mendelian predisposition to other mycobacterial infections in the context of the syndrome of Mendelian susceptibility to mycobacterial diseases (MSMD) and severe tuberculosis of childhood [9–12]. In addition, recent studies have reported association between BU and variants located in genes already implicated in MSMD, TB or leprosy [13, 14]. The role of host genetics in BU is further supported by two studies in Ghana [15] and in 11 African endemic countries including Benin [16] reporting highly restricted genetic variation of the microbe after the sequencing of more than 150 isolates of *M*. *ulcerans* therefore ruling out a major role of specific *M*. *ulcerans* strains in BU clinical outcomes. In the present work, we report an extensive genome-wide study aiming to decipher the genetic basis of a remarkably severe form of BU segregating in a large consanguineous multiplex family from Benin and suggestive of autosomal recessive inheritance.

## Materials and methods

### Familial data

The family was enrolled at the Centre de Dépistage et de Traitement de la Lèpre et de l’Ulcère de Buruli (CDTLUB) in Pobe, Benin, due to the unusual severity of the clinical course of the disease in patient P1 (details given in the case report section). The family consisted of two unaffected parents, two affected children and seven unaffected siblings living in the village of Tatonnonkon. This village is located in Adja-Ouèrè, a district of the Plateau department at the borders of the Zou and Ouémé departments, with BU prevalence ranging from 10 to 18 per 1,000 [17]. Blood was obtained from the parents, the two affected and three unaffected siblings. DNA was extracted from whole blood according to the Nucleon BACC2 Genomic DNA extraction protocol (GE Healthcare), assayed with the QuantIt Picogreen dsDNA kit (Life Technologies) and processed for the genotyping of >900,000 single nucleotide polymorphisms (SNPs) used for both linkage and copy number variant (CNV) analysis and >900,000 monomorphic nucleotides used for CNV analysis by the Affymetrix Genome-Wide 6.0 array.

The genetic research on susceptibility to BU was approved by the institutional review board of the CDTLUB and the national Beninese BU control authorities (IRB00006860), as well as the ethics committee of the university hospital of Angers, France (Comité d’Ethique du CHU d’Angers). All participants (parents and five children) provided written informed consent or had their parents provide written informed consent on their behalf. Parents of patients P1 and P2 have given written informed consent to publish anonymized case details including pictures and X-rays.

### Quality control for the genotyping data

Stringent quality control (QC) procedures were applied. Individual QC consisted of checking the individual call rate (>95%), the match between genetic and declared sex and the match between genetic and declared degrees of familial relatedness. For linkage analysis, family-based SNP QC was performed and only SNPs with a within-family SNP call rate of 100%, a non-zero minor allele frequency (MAF) and no Mendelian errors were retained. As several QC measures (e.g. Hardy-Weinberg equilibrium filters) cannot be applied to a single family because of its intrinsically limited size, we further filtered SNPs on a population basis, using the Yoruba population from the Hapmap project (Affymetrix 6.0 genotyping, [18]). We retained SNPs with a population call rate ≥95% and a non-zero MAF that were in Hardy-Weinberg equilibrium at the 0.01 level.

### Linkage analysis

Linkage analysis was performed by homozygosity mapping, a powerful statistical approach for detecting genetic linkage in the presence of familial consanguinity. We used MERLIN version 1.2 and its clustering option to take linkage disequilibrium into account and specified a recessive model with complete penetrance [19, 20]. Linkage regions were further screened for causal point mutations and/or structural variations, through whole-exome sequencing and CNV analysis. We also estimated homozygosity rates in children using runs of homozygosity as previously described [19, 20].

### Whole-exome sequencing

Whole-exome sequencing was performed on both affected patients. Genomic DNA was sheared with a Covaris S2 Ultrasonicator (Covaris). An adaptor-ligated library was prepared with the Paired-End Sample Prep kit V1 (Illumina). Exome capture was performed with the SureSelect Human All Exon v2 kit (Agilent Technologies), covering 38 Megabases (Mbs) of the genome. Single-end sequencing was performed on an Illumina Genome Analyzer IIx. The sequences were aligned with the human genome reference sequence (hg19/GRCh38 build), with BWA-MEM aligner [21]. Downstream processing was carried out with the Genome Analysis Toolkit (GATK) [22], SAMtools [23], and Picard Tools (http://broadinstitute.github.io/picard/). Substitution and indel calls were both made with GATK HaplotypeCaller v3.3. All calls with a Phred-scaled quality ≤30 were filtered out. Variant annotation was based on the Human genome assembly GRCh38 as implemented in the Ensembl browser (release 88) as previously described [24–26].

### CNV analysis

Genome-wide CNV analysis of the familial data was performed on Affymetrix 6.0 data with the joint calling algorithm of PennCNV, which takes familial information into account, to improve CNV calls within families [27]. In addition, we took advantage of an ongoing genetic study including 401 healthy local controls (Median age at the time of enrolment = 40 years old; Male:female sex ratio = 0.72) and 402 local laboratory-confirmed BU cases (Median age at the diagnosis = 11 years old; Male:female sex ratio = 0.81) genotyped with the Illumina Omni2.5 chip (which includes over 2.3 million SNPs) to further investigate any CNV-related findings made in the familial study. CNV analysis among these 803 individuals was performed as in the familial data by means of PennCNV. These 803 individuals of Yoruba ethnicity were also enrolled through the CDTLUB in Pobe, Benin, and lived in villages distributed over the Ouémé and the Plateau departments in an endemic area with BU prevalence estimated around 8 to 20 per 1,000 [17, 28].

### Refined targeted sequencing

Refined targeted resequencing by Next-Generation Sequencing (NGS) to detect CNVs was performed in four individuals of the family (the two affected sisters predicted to be homozygous for the deletion by PennCNV, one predicted heterozygote brother and one predicted homozygous wild-type brother) and six independent controls (2 men and 4 women all predicted to be homozygous wild-type) that were part of the 401 local controls described above and aged above 30 years old to increase the likelihood of exposure to *M. ulcerans*. Targeted resequencing was done by means of ‘capture by hybridization’ approach. Illumina compatible bar-coded genomic DNA libraries were constructed according to the manufacturer’s sample preparation protocol (Ovation Ultralow, Nugen Technologies). Briefly, 1 to 3 μg of each patient’s genomic DNA was mechanically fragmented to a median size of 200 base pairs (bps) using a Covaris. 100 ng of double strand fragmented DNA was end-repaired and adaptors containing a specific eight bases bar-code were ligated to the repaired ends (one specific bar-code per patient). DNA fragments were PCR amplified to get the final precapture bar-coded libraries that were pooled at equimolar concentrations (a pool of 15 libraries was prepared). The biotinylated single strand DNA probes were designed and prepared to cover a 157 kilobases (kbs) chromosomal region on chromosome 8. The limits of the targeted chromosomal region are Chr8:12,532,612–12,690,151 according to the GRCh38 assembly of the human reference genome. During the capture process, bar-coded libraries molecules complementary to the biotinylated beads were retained by streptavidin coated magnetic beads on a magnet and PCR amplified to generate a final pool of post capture libraries covering the targeted chromosomal region on chromosome 8. In total a pool of 15 libraries covering the 157 kbs of interest on chromosome 8 was sequenced on an Illumina HiSeq2500 (Paired-End sequencing 130x130 bases, High Throughput Mode, 15 samples per lane). Finally, sequence reads were aligned to the human hg19/GRCh38 reference genome using the Burrows-Wheeler Alignment version 0.6.2.13 [21].

## Results

### Case report

**Fig 1:**
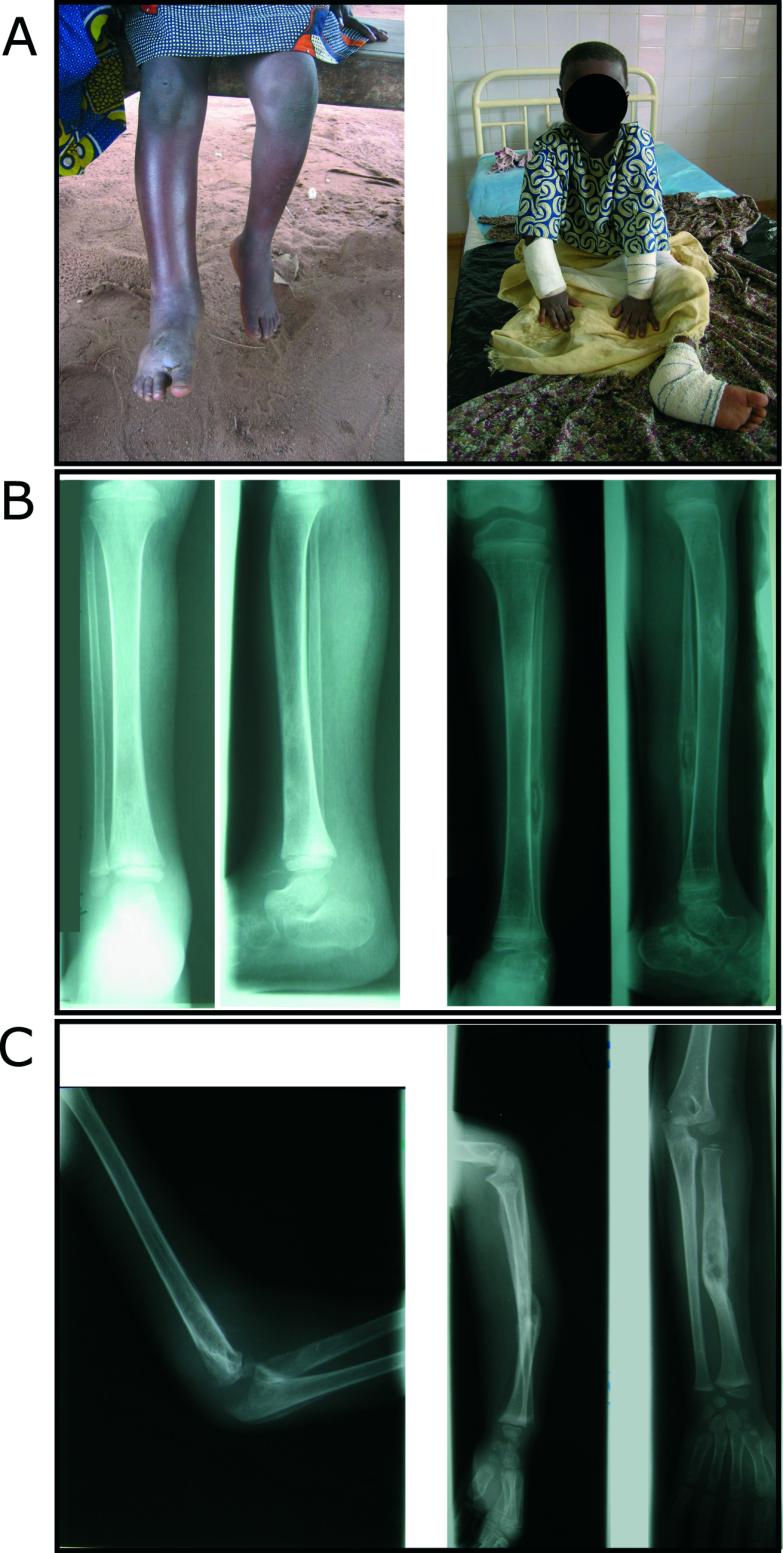
Clinical course of unusually severe BU in the index patient P1. All four limbs were affected by edematous BU lesions, with osteomyelitis involving ten bones and septic arthritis involving two joints. (A) Severe edema of the right leg and foot and edema of the left foot (left panel) leading to amputation of the lower right leg (right panel). (B) Mediodiaphyseal lacuna of the lower third of the right tibia and major soft tissue enlargement (left panel). Hypercondensation of the proximal third of the left tibia and lacuna of the lower third of the fibular diaphysis with central sequestrum (right panel). (C) Intra-articular effusion of the right elbow associated to osteolysis and periostal reaction of the humerus, the olecranon and the radial head (left panel). Forearm diaphyseal lacuna of the left radius with sequestrum and partly consolidated fracture of the left radius (right panel).

The index case, P1, a girl born in 2000, was identified as the most severe case of BU ever diagnosed among more than 2,000 patients seen at the CDTLUB in Pobe, Benin since its opening in 2003 (Fig 1 and S1 Fig). The parents were self-declared second-degree cousins. At the age of five years, P1 presented with cachexia, fever and an edematous lower right limb (Fig 1A, left pannel). *Mycobacterium ulcerans* was identified in the synovial fluid of the right knee by Ziehl-Neelsen staining and IS2404 PCR amplification. P1 also presented with PCR-positive edema of the left foot, leading to the X-ray confirmed diagnosis of osteomyelitis of the left cuboid and osteoarthritis of the right tibia and knee (Fig 1B, left panel). Despite early and prolonged (13 weeks vs. 8 weeks for the standard regimen) antibiotic treatment with rifampicin and streptomycin and several surgical procedures, the infection spread further. Two months after diagnosis, edema of the right arm revealed *M*. *ulcerans* osteoarthritis of the right elbow, further confirmed by the aspiration of caseiform matter, Ziehl-Neelsen staining and PCR amplification (Fig 1C, left panel). Sustained fever did not recede before the amputation of the patient’s right leg after three months (Fig 1B, right panel). After six months, osteomyelitis of the left radius was diagnosed (Fig 1C, right panel). One year after diagnosis, left fibula involvement was detected (Fig 1B, right panel).

P1 thus suffered from unusually severe edematous BU with unprecedented dissemination to ten bones and two joints in all four limbs (Fig 1 and S1 Fig). She underwent a number of surgical procedures on all four limbs in the first 18 months after diagnosis. The disease relapsed two, three and five years after the initial diagnosis, with the re-emergence of infection at new sites: the right humerus, left calcaneal tendon and left tibia, respectively (S1 Fig). *Mycobacterium ulcerans* was repeatedly identified by Ziehl-Neelsen staining and IS2404 PCR on the various lesions of all four limbs, including surgical bone samples, at different time points (S1 Fig). The medical history of P1 also included uncomplicated acute HBV hepatitis and several episodes of classical malaria up to 2016. However, as of December 2017, no additional BU-related clinical events have been reported.

P1’s sister, P2, was born in 1995. At 13 years of age, she was also diagnosed with a severe form of BU, presenting as rapidly spreading edema of the whole right arm, right forearm and right hand and a large plaque of the right elbow. PCR and culture were positive for *M*. *ulcerans*. She was given 8 weeks of antibiotic treatment combining rifampicin and streptomycin. Over the three months following the diagnosis, she underwent several excision and curettage surgeries and a skin graft (S1 Fig). Although diagnosed very rapidly after clinical expression of the disease due to the strict clinical supervision of P1 and her family, P2 still presented with a severe form including large edema and plaque. Of particular interest is the observation that the two patients presented a severe non-ulcerative form of BU, a rare occurrence in the clinical landscape of the disease [29]. As easily inferable, P1 and P2 suffer from permanent functional limitations significantly impacting their daily life. They were HIV-negative and had been vaccinated with BCG at birth. Their complete blood formulas were normal. On the contrary, both parents and seven other siblings were unaffected at the time of the study and to the best of our knowledge still were as of December 2017.

### Genetic investigations

After QC, 310,993 SNPs of the Affymetrix 6.0 array remained and were grouped into 126,704 independent clusters for model-based linkage analysis by homozygosity mapping, reaching a genome-wide mean information content of 0.99, based on genotyped individuals. Identity-by-state analysis confirmed that P2’s twin was a fraternal, not identical, twin. Homozygosity was estimated at ~2% in the five children of the family for whom DNA was available, consistent with the level of self-declared consanguinity, i.e. second degree cousin for the parents [30]. Homozygosity mapping aimed at identifying regions of shared runs of homozygosity inherited identical-by-descent by affected individuals, but not by unaffected individuals. We computed the theoretical maximum LOD score at a completely informative marker, given the familial configuration and the genetic model. In our scenario (two affected siblings, three unaffected siblings, consanguinity loop involving second-degree cousins and a recessive genetic model with complete penetrance), this theoretical maximum LOD score was equal to 2.8 (corresponding to a p-value of 3x10^-4^).

**Fig 2:**
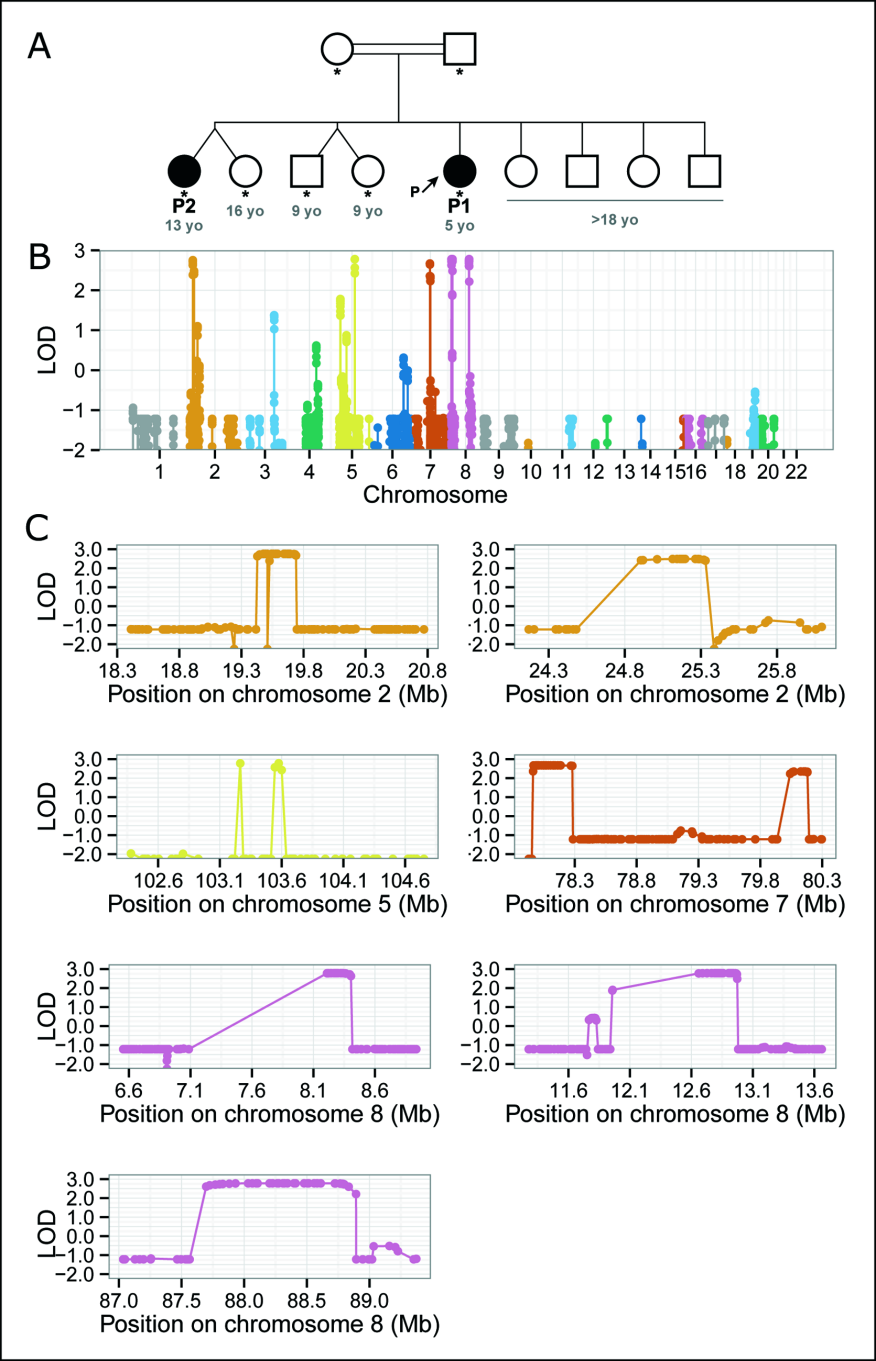
BU of unusual severity segregates in a consanguineous family from Benin and maps to chromosomes 2, 5, 7 and 8. (A) Pedigree tree. Genotyped individuals are indicated by stars with age in years (blue numbers) given at the time of diagnosis for the two patients and at the time of the study for unaffected individuals. Two of the nine siblings were affected and the parents were consanguineous. We therefore hypothesized a recessive mode of inheritance, and carried out linkage analysis by homozygosity mapping. Note that P2’s twin is not an identical twin. (B) Genome-wide linkage analysis by homozygosity mapping. This statistical approach aims to identify genomic regions homozygous and identical-by-descent in affected individuals but not homozygous in unaffected individuals. The evidence for linkage is based on LOD scores (y axis). (C) Linkage regions on chromosomes 2 (two regions: 2.1, 2.2), 5, 7 (7.1, 7.2), and 8 (8.1, 8.2, 8.3). Homozygosity mapping identifies 8 linkage regions spanning hundreds of kbs to Mbs very likely to contain the causal genetic lesion but does not identify the genetic lesion itself. For the exact coordinates of the linkage regions, see S1 Table.

Eight regions spanning a total of 5.7 Mbs and mapping to chromosomes 2 (linkage regions 2.1 and 2.2), 5, 7 (7.1 and 7.2) and 8 (8.1, 8.2 and 8.3) reached or closely approached the theoretical maximum LOD score ((Fig 2). The second region on chromosome 2 (2.2), and both regions on chromosome 7 (7.1 and 7.2) had LOD score of 2.49, 2.68 and 2.35, respectively. All the other linked regions had a LOD score above 2.75. The complete list of genes in each of these regions, retrieved from the Vega database as implemented in the Ensembl browser (vega.archive.ensembl.org), is available in the S1 Table. Interestingly, two of the three linkage regions on chromosome 8, i.e. 8.1 and 8.2, contain clusters of genes encoding beta-defensins (S2 Fig).

We screened the linkage regions for potential causal mutations by means of whole-exome sequencing of the two patients. We searched for homozygous variants predicted to have potential functional effect, i.e. missense, nonsense and splice site mutations, in-frame and out-of-frame small insertions and deletions, common to both patients and located in the linkage regions. We filtered out population variants with a MAF above 1% from public databases such as the Exome Aggregation Consortium (ExAC; exac.broadinstitute.org). No candidate variants fitting these criteria were detected. However, the two linkage regions harboring clusters of beta-defensin genes (i.e. 8.1 and 8.2) displayed low mapping quality with mean values of 2 and 11, respectively, versus values >55 for all other linkage regions, and a mean mapping quality value of 46 for the whole-exome.

**Fig 3:**
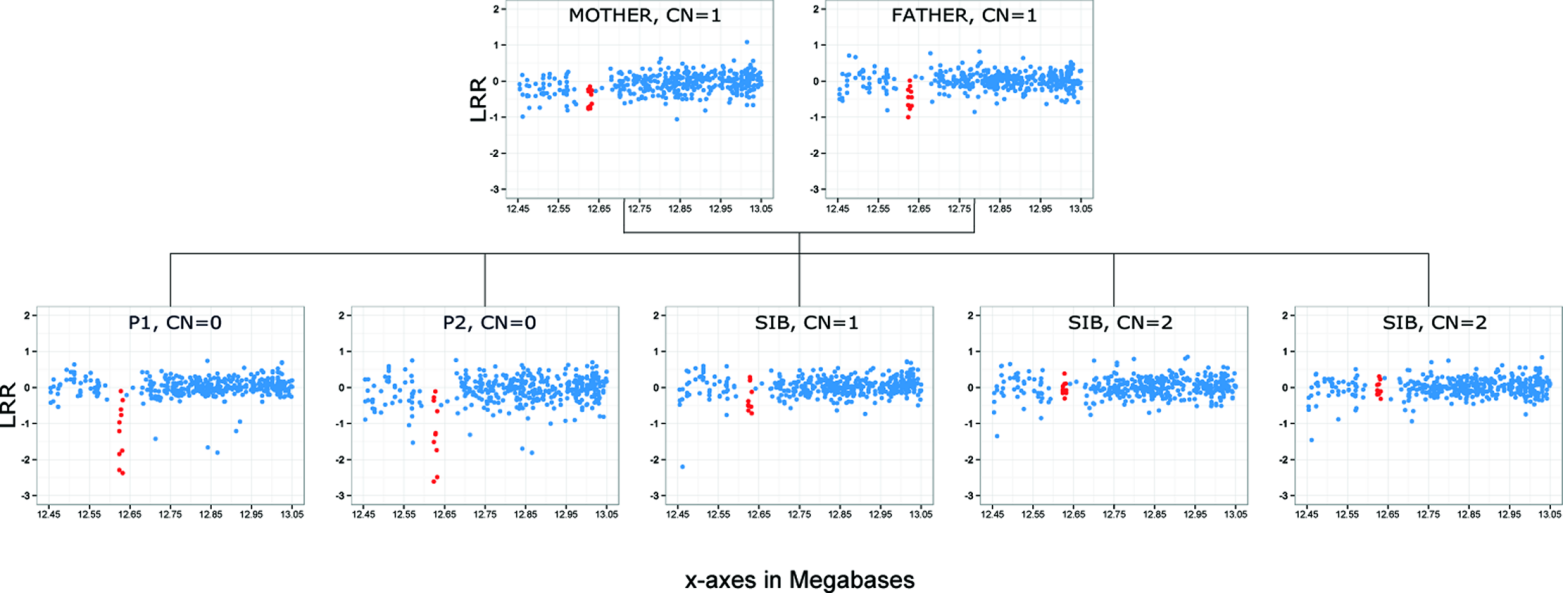
Genome-wide CNV analysis identifies a homozygous deletion in the second linkage region on chromosome 8. CNV identification is based principally on quantitative analysis of the intensity of the hybridization signal of nucleotide probes (the logR ratio, LRR) throughout the genome, making it possible to infer that the copy number (CN) is normal, increased or decreased in the patient’s genome, at the location of the probe. Each blue dot represents one probe in this region of chromosome 8. Red dots represent the CNV segment identified in this family by PennCNV (from probe CN_1273661 to probe CN_1273690, at position 12,616,022 to 12,624,550 in GRCh38 coordinates).

Next, we screened the linkage regions for structural variations by means of PennCNV analysis of the seven members of the family [27]. A single homozygous deletion common to both patients was found around position 12,616,035 on chromosome 8 (GRCh38 assembly). The deletion was predicted to be heterozygous in both parents, heterozygous in one unaffected sibling and absent from the other two unaffected siblings studied therefore co-segregating perfectly with the phenotype (Fig 3). This deletion was located close to a cluster of beta-defensin genes in linkage region 8.2 (S2 Fig) and was the only CNV, anywhere in the genome, to display this perfect pattern of familial segregation. The genetic screening of 401 local controls by means of PennCNV (see Materials and Methods section) identified two individuals heterozygous for the deletion but did not detect any homozygous carriers. Based on these results, the frequency of the deletion was predicted to be 2.5x10^-3^. Of note, CNV prediction algorithms based on genotyping data may not have an optimal resolution to detect heterozygous carriers of small deletion, i.e. true frequency for the deletion may be somewhat higher. The same screening of 402 local BU cases identified two homozygous carriers of the deletion: one female diagnosed at the age of 25 years old with a severe form of BU, i.e. 20 centimeters edema located on the left lower limb therefore classified as 3 in the 3-class WHO severity scale, and another female diagnosed at the age of 15 years old with a less severe form, i.e. a 12 centimeters edema located on the lower limb therefore classified as 2 according to WHO scale. Remarkably, similar to P1 and P2 these two patients also developed a non-ulcerative form of the disease.

**Fig 4:**
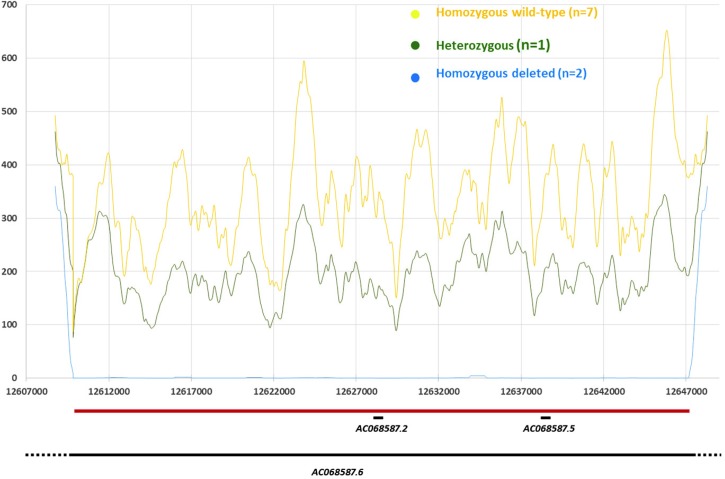
Distribution of the mean coverage per base in the 8q32 deletion region according to the deletion status. Zoom out of the deletion region (Chr8:12,607,000–12,647,000). Positions in base pair (bp) are given on the x-axis. Y-axis displays the mean coverage per base as estimated through 1kb sliding windows (i.e. value at position X is the mean over X +/- 500 bp; note for X<1kb mean is over X + 1kb). Custom tracks shows the delineated deletion (Chr8:12,609,841–12,647,341—horizontal red bar), and the genes reported from the Vega database as implemented in the Ensembl genome browser (horizontal black lines): *AC068587*.*6 (*Ensembl gene ID: *ENSG00000283674)*; *AC068587*. *2 (ENSG00000244289); AC068587*.*5 (ENSG00000255253)*.

We performed targeted resequencing of the CNV region by original NGS experiments, i.e. capture by hybridization approach, the most accurate approach to date to detect CNV in the context of our study. This resequencing was performed in four individuals of the family (the two affected sisters predicted to be homozygous for the deletion by PennCNV, one predicted heterozygous brother and one predicted homozygous wild-type brother) and six independent controls (all predicted to be homozygous wild-type) that were part of the 401 local controls and aged above 30 years old (Fig 4). The distribution of the mean number of reads (X) in the targeted region allowed us to validate unambiguously the deletion at the molecular level, and to refine its breakpoints from chr8:12,609,841 to chr8:12,647,341 (GRCh38 assembly). Over this 37 kbs region the mean (interquartile range) coverage was 0.47 X (0–0) in the two affected sisters homozygous for the deletion, 198.5 X (94–274) in the unaffected heterozygote brother and 342.8 X (196–515) in the seven wild-type homozygous controls (including one unaffected brother with a mean coverage of 330.1 X).

A brief description of this region–derived from the Vega database as implemented in the Ensembl genome browser (vega.archive.ensembl.org)–based on GRCh38 Assembly is given in Fig 4. Three genes of different biotypes have been identified so far including two processed pseudogenes *AC068587*.*5* (Ensembl id: *ENSG00000255253*; spanning 174bp from 12,638,428 to 12,638,602) and *AC068587*.*2* (*ENSG00000244289*; 780bps from 12,628,476 to 12,629,256), and one long non-coding RNA (lincRNA) gene *AC68587*.*6* (*ENSG00000283674*; ~128kbs from12,537,079 to 12,665,588). Remarkably, this lincRNA gene was previously shown to display highest expression values in the skin (https://www.ncbi.nlm.nih.gov/gene/?term=ensg00000283674) [31].

Overall, the observations that 1) exome sequencing found no evidence for a variant with potential functional effect in the genes of the linked regions, 2) the confirmed deletion co-segregates with the phenotype in our family with P1 and P2 being homozygous carriers for the deletion, 3) no homozygous carriers were found in a sample of 402 local controls, 4) two homozygous carriers were found in a sample of 401 local BU cases and 5) the deletion includes a lincRNA with highest expression values in the skin and located close to a cluster of beta-defensin genes, altogether suggest this deletion as a critical trigger of severe non-ulcerative forms of BU.

## Discussion

We report the clinical phenotype and extensive genetic analysis of a multiplex consanguineous family with BU of unusual severity displaying Mendelian inheritance. The index patient P1 suffered from aggressive multifocal edematous BU and *M*. *ulcerans* osteoarthritis with several relapses over a period of five years despite intensive medical and surgical care. The disease spread to all four limbs and resulted in severe handicap, including amputation of the right leg. P1’s sister also suffered from severe edematous BU of the right arm, forearm and hand, leading to permanent functional sequelae. The subsequent genetic investigations were performed assuming P1 and P2 should share the same genetic defect because they share similar phenotypes. This assumption may sound somewhat speculative as the clinical picture of P1 appeared more severe than the one of P2. However, two important aspects must be considered. First, because of P1 dramatic clinical course, the whole family was under tight clinical monitoring, and, although diagnosed at a significantly earlier stage than P1, P2 still presented a severe form of BU. Any delay in the diagnosis may have resulted in a more severe clinical outcome. Second, P1 and P2 developed a very severe *non-ulcerative* form of the disease, a rare clinical picture first emphasized in 2000 [29].

We searched for homozygous mutations located within these linkage signals, by performing whole-exome sequencing in both patients. We did not identify any homozygous mutations common to the two affected sisters within the linkage regions. We could not exclude the possibility of such mutations being present in the two beta-defensin-containing linkage regions 8.1 and 8.2, as the most recent alignment algorithms fail to generate a unique alignment of the sequencing reads in the beta-defensin clusters [32]. Extremely high levels of sequence repetition resulted in very low mapping quality values in these regions, precluding reliable genotype calling. We checked both public and in-house whole-genome sequencing data generated from other individuals and found that this sequencing method, which generally performs better overall, also suffered from this limitation in the defensin region [33]. The ongoing development of novel sequencing technologies based on the generation of reads as long as several Mbs [34] should provide an efficient mean to overcome this issue. Nevertheless, our analysis of whole-exome sequencing data for both patients in regions that were well-covered with excellent mapping quality (i.e. the large majority of exome target sequences) was useful to rule out the presence of homozygous candidate mutations in this family.

Because whole-exome sequencing did not provide any evidence for exonic point mutations explaining the linkage signals, we tested the hypothesis of structural variations as potential causes of these signals by means of *in silico* CNV analysis. We found a homozygous deletion spanning ~ 10 kbs located within the linkage region 8.2 common to P1 and P2 but absent from unaffected siblings and 401 local controls. Further screening of 402 unrelated BU local cases identified two additional homozygous carriers. Remarkably, the two homozygous carriers of the deletion also developed a non-ulcerative form of the disease. This is suggestive of severe non-ulcerative BU being a very specific clinical form of the disease. CNV detection algorithms have a modest resolution for determining CNV breakpoints and are known to underestimate CNV size, implying that the deletion identified here may be larger [35]. Indeed, targeted resequencing in 10 individuals (including four siblings and six local controls) confirmed the presence of a homozygous 37 kbs deletion in the two patients, heterozygous in one unaffected sib and absent in the remaining individuals. Of note, all *in silico* predictions were unambiguously confirmed by the resequencing assay further supporting the *in-silico* results observed in the local sample of 401 controls and 402 cases.

The deletion extends from chr8:12,609,841 to chr8:12,647,341 and comprises the lincRNA gene *AC068587*.*6* located in close vicinity to beta-defensin clusters. LincRNAs are thought to regulate the expression of neighboring genes with very strong tissue specificity [36]. Remarkably, *AC068587*.*6* has been shown to display its highest expression values in the skin ((https://www.ncbi.nlm.nih.gov/gene/?term=ensg00000283674). In addition, it is located close to a cluster of beta-defensins encoding genes (S1 Table). Beta-defensins are a family of antimicrobial peptides involved in innate immunity; they are widely secreted, throughout epithelial tissues, in response to infectious agents [37–40]. Interestingly, beta-defensins have also been implicated in the healing process of aseptic skin wounds [37, 39–41]. The antibacterial and wound repair functions of beta-defensins are consistent with a role for these molecules in the human response to *M*. *ulcerans*. They have been shown, *in vitro* and *in vivo*, to be upregulated in response to several mycobacteria [42–47], including *M*. *ulcerans* [48], and have been specifically implicated in the response to bone infection in both mice and humans [49–51]. These biological functions of beta-defensins together with the highest expression of *AC068587*.*6* in skin, likely the most relevant tissue in BU pathophysiology, strongly support the hypothesis of this lincRNA playing a role in the pathophysiology of BU.

Most of the exposed individuals in foci of highly endemic infection do not develop BU lesions, but some, such as P2, rapidly develop extensive skin lesions, and others, such as P1, rapidly develop multifocal skin and bone lesions despite aggressive medical and surgical treatment. This inter-individual variability in the human response to *M*. *ulcerans* may have a genetic origin, a hypothesis supported by the identification of a homozygous deletion within a linkage region containing beta-defensin genes in this large multiplex family from Benin. A number of monogenic predispositions to common infections, such as tuberculosis, have been described and genetically dissected over the last decade [9–12, 52–57] but this is the first report of monogenic inheritance in severe BU. More refined functional investigations, such as silencing RNA followed by defensins dosage, are needed to obtain additional sources of evidence supporting causality between the deletion identified, the lincRNA, beta-defensins and the clinical phenotype [58]. We will need to face up to the non-trivial difficulties posed by the extreme complexity of this repetitive and dynamic region of the human genome, an exciting and promising challenge for future research.

## Supporting information

S1 Fig Detailed clinical course of the two severe BU patients P1 and P2. (PDF)

S2 Fig Schematic representation of beta-defensin clusters located in linkage regions 1 and 2 on chromosome 8. (PDF)

S1 Table List of genes in the eight linkage regions retrieved from the Vega database as implemented in the Ensembl browser (vega.archive.ensembl.org). (PDF)

S1 List Franco-Beninese Buruli Research Group. List of participants according to their geographic location.
(PDF)
