# Supplementary material for: Microdeletion on chromosome 8p23.1 in a familial form of severe Buruli ulcer
Source: PLoS Negl Trop Dis. 2018 Apr 30;12(4):e0006429. doi: 10.1371/journal.pntd.0006429 (PMC5945055; doi:10.1371/journal.pntd.0006429)
Supplement: S1 Fig — (PDF) [file pntd.0006429.s001.pdf]

**S1 Figure. Detailed clinical course of the two severe BU patients P1 and P2.**

## Patient P1

| YEAR | | CLINICAL EVENTS and TREATMENT |
|---|---|---|
| 2000 | | **Birth** |
| 2005 | **5 yo** | **Initial Diagnosis**<br>-Edema: right thigh&leg and left foot + Septic arthritis: right knee<br>-Osteomyelitis : right fibula and tibia; left cuboid<br>-Lab: Ziehl-Neelsen and PCR+ on both limbs<br>*Rifampicin + streptomycin (13 weeks)*<br><br>***Dissemination (2 months post-diagnosis)***<br>-Edema: right arm and forearm + Septic arthritis: right elbow<br>-Osteomyelitis : right humerus and radius and ulna<br>-Lab: Ziehl-Neelsen and PCR +<br>Amputation of the lower right limb (3 months post-diagnosis)<br><br>***Dissemination (6 months post-diagnosis)***<br>-Edema: left forearm+ Osteomyelitis : left radius<br>-Lab: Ziehl-Neelsen +; PCR not performed<br>Numerous surgeries |
| 2006 | 6 yo | ***Dissemination (1 year post-diagnosis)***<br>-Edema: left leg + Osteomyelitis : left fibula<br>-Lab: Ziehl-Neelsen and PCR +<br>Numerous surgeries |
| 2007 | 7 yo | ***Dissemination (2 years post-diagnosis)***<br>-Edema: right arm + Osteomyelitis : right humerus<br>-Lab: Ziehl-Neelsen +; PCR not performed<br>Numerous surgeries |
| 2008 | 8 yo | ***Dissemination (3 years post-diagnosis)***<br>-Soft tissue collection: left calcaneal tendon<br>Surgery |
| 2010 | 10 yo | ***Dissemination (5 years post-diagnosis)***<br>-Edema: left leg + Osteomyelitis : left tibia<br>-Lab: Ziehl-Neelsen and PCR +<br>Surgery |

No reported BU-related events
(as of 12/2017)

## Patient P2

| MONTH/YEAR | | CLINICAL EVENTS and TREATMENT |
|---|---|---|
| 1995 | | **Birth** |
| 12/2008 | **13 yo** | **Initial Diagnosis**<br>- Large Edema: right arm, right forearm and right hand<br>- Plaque : right elbow<br><br>*Rifampicin + streptomycin (8 weeks)* |

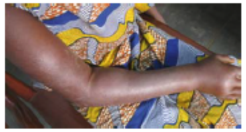

| MONTH/YEAR | | CLINICAL EVENTS and TREATMENT |
|---|---|---|
| 01/2009 | | *Partial excision of the plaque (16/01)*<br>*Additional excision and curretage (20/01)* |
| 02/2009 | | *Aditionnal excision and curretage (17/02)*<br>*Skin graft (26/02)* |
| 04/2009 | | *discharge* 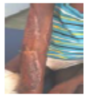 |
| 10/2009 | **14 yo** | *Surgery: pedicle flap of the elbow* |

No reported clinical event
(as of 12/2017)
