# Supplementary material for: Microdeletion on chromosome 8p23.1 in a familial form of severe Buruli ulcer
Source: PLoS Negl Trop Dis. 2018 Apr 30;12(4):e0006429. doi: 10.1371/journal.pntd.0006429 (PMC5945055; doi:10.1371/journal.pntd.0006429)

S2 Figure. Schematic representation of beta-defensin clusters located in linkage regions 1 and 2 on chromosome 8.

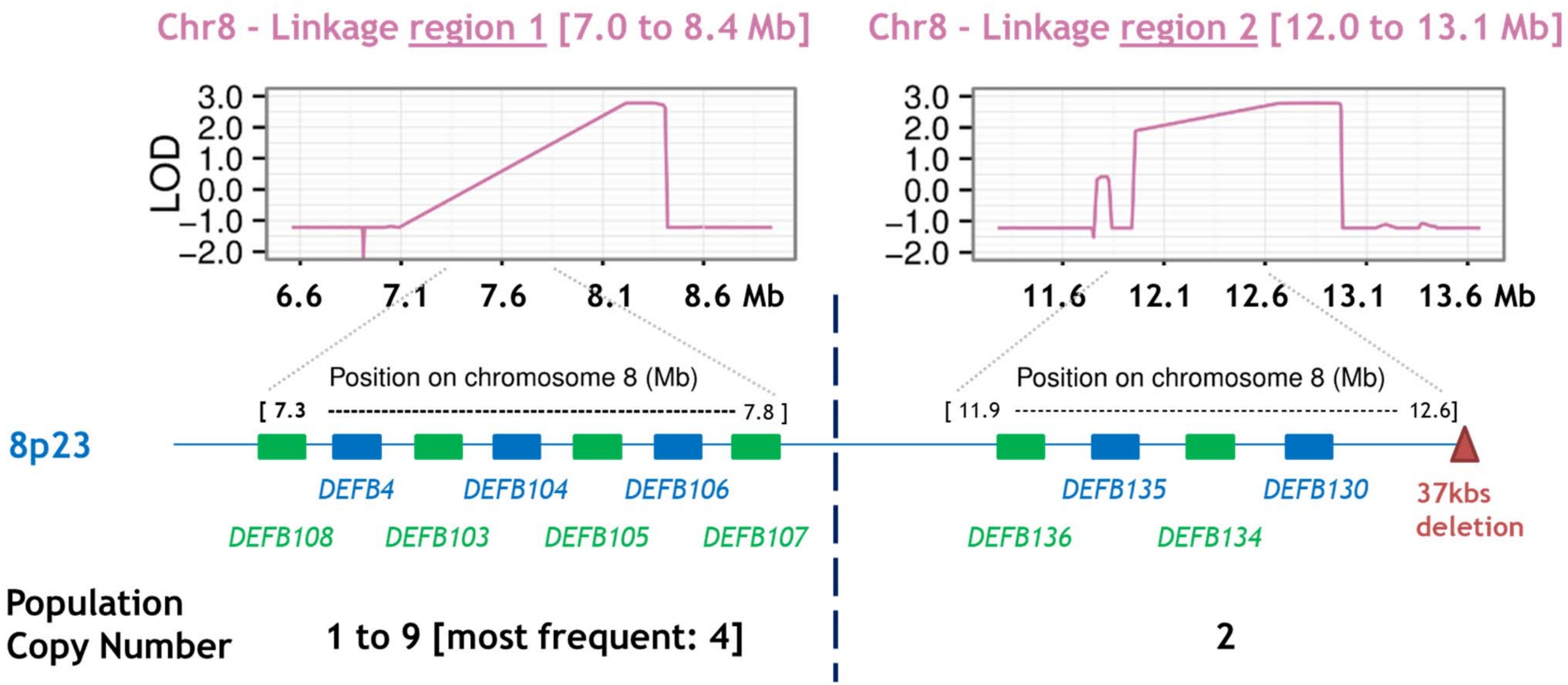

Supplement: S2 Fig — (PDF) [file pntd.0006429.s002.pdf]
