# Supplementary material for: Microdeletion on chromosome 8p23.1 in a familial form of severe Buruli ulcer
Source: PLoS Negl Trop Dis. 2018 Apr 30;12(4):e0006429. doi: 10.1371/journal.pntd.0006429 (PMC5945055; doi:10.1371/journal.pntd.0006429)
Supplement: S1 Table — (PDF) [file pntd.0006429.s003.pdf]

S1 Table. List of genes in the eight linkage regions retrieved from the Vega database as implemented in the Ensembl browser (vega.archive.ensembl.org).

Chrom.linkloc	Gene start (bp)	Gene end	Gene ensembl ID	Gene biotype	Gene name	Gene description
2.1	19458220	19468961	ENSG00000234597	lincRNA	AC010096.1	
2.1	19475450	19488850	ENSG00000237992	lincRNA	LINC01808	long intergenic non-protein coding RNA 1808 [HGNC:52611]
2.1	19711715	19717576	ENSG00000235911	lincRNA	AC019055.1	
2.2	24491914	24770702	ENSG00000084676	protein_coding	NCOA1	nuclear receptor coactivator 1 [HGNC:7668]
2.2	24564630	24564738	ENSG00000202430	rRNA	RNA5SP88	RNA, 5S ribosomal pseudogene 88 [HGNC:42886]
2.2	24676309	24676415	ENSG00000206732	snRNA	RNU6-936P	RNA, U6 small nuclear 936, pseudogene [HGNC:47899]
2.2	24789734	24793382	ENSG00000184924	protein_coding	PTRHD1	peptidyl-tRNA hydrolase domain containing 1 [HGNC:33782]
2.2	24793136	24822376	ENSG00000138092	protein_coding	CENPO	centromere protein O [HGNC:28152]
2.2	24819169	24919839	ENSG00000138031	protein_coding	ADCY3	adenylate cyclase 3 [HGNC:234]
2.2	24825610	24826717	ENSG00000271936	antisense	RP11-443B20.1	
2.2	24943636	24972094	ENSG00000115137	protein_coding	DNAJC27	DnaJ heat shock protein family (Hsp40) member C27 [HGNC:30290]
2.2	24968958	24969224	ENSG00000237953	processed_pseudogene	AC013267.1	
2.2	24969388	24969477	ENSG00000202479	snoRNA	SNORD14	Small nucleolar RNA SNORD14 [Source:RFAM;Acc:RF00016]
2.2	24971390	25039694	ENSG00000224165	antisense	DNAJC27-AS1	DNAJC27 antisense RNA 1 [HGNC:42943]
2.2	25008845	25008946	ENSG00000207069	misc_RNA	Y_RNA	Y RNA [Source:RFAM;Acc:RF00019]
2.2	25042130	25159137	ENSG00000084710	protein_coding	EFR3B	EFR3 homolog B [HGNC:29155]
2.2	25058032	25058329	ENSG00000276653	misc_RNA	RN7SL856P	RNA, 7SL, cytoplasmic 856, pseudogene [HGNC:46872]
2.2	25079901	25081689	ENSG00000229593	processed_pseudogene	SUCLA2P3	succinate-CoA ligase ADP-forming beta subunit pseudogene 3 [HGNC:38103]
2.2	25160853	25168903	ENSG00000115138	protein_coding	POMC	proopiomelanocortin [HGNC:9201]
2.2	25204313	25209202	ENSG00000230452	antisense	LINC01381	long intergenic non-protein coding RNA 1381 [HGNC:50653]
2.2	25227855	25342590	ENSG00000119772	protein_coding	DNMT3A	DNA methyltransferase 3 alpha [HGNC:2978]
2.2	25328640	25328721	ENSG00000221445	miRNA	MIR1301	microRNA 1301 [HGNC:35253]
5	103408941	103412511	ENSG00000250567	lincRNA	CTD-2154H6.1	
5	103430406	103526618	ENSG00000226926	transcribed_unitary_pseudogene	PDZPH1P	PDZ and pleckstrin homology domains 1, pseudogene [HGNC:51488]
5	103528434	103541985	ENSG00000248757	lincRNA	LINC02115	long intergenic non-protein coding RNA 2115 [HGNC:52970]
5	103548855	103562793	ENSG00000112874	protein_coding	NUDT12	nudix hydrolase 12 [HGNC:18826]
7.1	78017057	79453574	ENSG00000187391	protein_coding	MAGI2	membrane associated guanylate kinase, WW/PDZ domain containing 2 [HGNC:18957]
7.1	78134079	78134851	ENSG00000281008	TEC	RP4-587D13.1	
7.1	78170195	78170915	ENSG00000280958	TEC	RP4-587D13.2	
7.1	78347142	78359458	ENSG00000231322	transcribed_processed_pseudogene	RPL13AP17	ribosomal protein L13a pseudogene 17 [HGNC:36167]
7.2	80096600	80097215	ENSG00000230853	processed_pseudogene	RPL10P11	ribosomal protein L10 pseudogene 11 [HGNC:36565]
7.2	80133955	80219402	ENSG00000127955	protein_coding	GNAI1	G protein subunit alpha i1 [HGNC:4384]
7.2	80245926	80246225	ENSG00000244392	misc_RNA	RN7SL869P	RNA, 7SL, cytoplasmic 869, pseudogene [HGNC:46885]
7.2	80246409	80312456	ENSG00000234223	lincRNA	AC003988.1	
8.1	7143632	7143841	ENSG00000254683	processed_pseudogene	SNRPCP6	small nuclear ribonucleoprotein polypeptide C pseudogene 6 [HGNC:49821]
8.1	7170550	7170741	ENSG00000230106	processed_pseudogene	SNRPCP15	small nuclear ribonucleoprotein polypeptide C pseudogene 15 [HGNC:49830]
8.1	7190901	7191563	ENSG00000214268	processed_pseudogene	RPS3AP33	ribosomal protein S3a pseudogene 33 [HGNC:35476]
8.1	7200756	7200857	ENSG00000231930	processed_pseudogene	AF228730.5	
8.1	7238286	7238631	ENSG00000255025	processed_pseudogene	AF228730.13	
8.1	7246726	7247571	ENSG00000177306	unprocessed_pseudogene	OR7E125P	olfactory receptor family 7 subfamily E member 125 pseudogene [HGNC:15098]
8.1	7256904	7259914	ENSG00000230045	unprocessed_pseudogene	FAM90A15P	family with sequence similarity 90 member A15, pseudogene [HGNC:32263]
8.1	7264526	7267536	ENSG00000233132	unprocessed_pseudogene	FAM90A3P	family with sequence similarity 90 member A3, pseudogene [HGNC:32251]
8.1	7272148	7275158	ENSG00000249005	unprocessed_pseudogene	FAM90A4P	family with sequence similarity 90 member A4, pseudogene [HGNC:32252]
8.1	7279770	7282780	ENSG00000223885	unprocessed_pseudogene	FAM90A13P	family with sequence similarity 90 member A13, pseudogene [HGNC:32261]
8.1	7287392	7290402	ENSG00000215373	unprocessed_pseudogene	FAM90A5P	family with sequence similarity 90 member A5, pseudogene [HGNC:32253]
8.1	7295014	7298024	ENSG00000233295	unprocessed_pseudogene	FAM90A20P	family with sequence similarity 90 member A20, pseudogene [HGNC:32268]
8.1	7301611	7355354	ENSG00000215374	lincRNA	FAM66B	family with sequence similarity 66 member B [HGNC:28890]
8.1	7312846	7319951	ENSG00000206034	unprocessed_pseudogene	DEFB109P1B	defensin beta 109 pseudogene 1B [HGNC:33469]
8.1	7332387	7333979	ENSG00000230549	protein_coding	USP17L1	ubiquitin specific peptidase 17-like family member 1 [HGNC:37182]

Chrom.linkloc	Gene start (bp)	Gene end	Gene ensembl ID	Gene biotype	Gene name	Gene description
8.1	7337115	7338707	ENSG00000236125	protein_coding	USP17L4	ubiquitin specific peptidase 17-like family member 4 [HGNC:37176]
8.1	7341826	7342942	ENSG00000235778	unprocessed_pseudogene	AC130360.8	
8.1	7355517	7385558	ENSG00000215372	protein_coding	ZNF705G	zinc finger protein 705G [HGNC:37134]
8.1	7373114	7377500	ENSG00000215371	unprocessed_pseudogene	DEFB108P2	defensin beta 108 pseudogene 2 [HGNC:30847]
8.1	7414860	7416863	ENSG00000177257	protein_coding	DEFB4B	defensin beta 4B [HGNC:30193]
8.1	7418991	7420546	ENSG00000255128	processed_pseudogene	HSPD1P3	heat shock protein family D (Hsp60) member 1 pseudogene 3 [HGNC:5264]
8.1	7428888	7430348	ENSG00000177243	protein_coding	DEFB103B	defensin beta 103B [HGNC:31702]
8.1	7442684	7463674	ENSG00000164871	protein_coding	SPAG11B	sperm associated antigen 11B [HGNC:14534]
8.1	7470308	7475082	ENSG00000177023	protein_coding	DEFB104B	defensin beta 104B [HGNC:26165]
8.1	7482504	7486400	ENSG00000187082	protein_coding	DEFB106B	defensin beta 106B [HGNC:28879]
8.1	7487669	7489593	ENSG00000186599	protein_coding	DEFB105B	defensin beta 105B [HGNC:29930]
8.1	7495846	7509311	ENSG00000198129	protein_coding	DEFB107B	defensin beta 107B [HGNC:31918]
8.1	7539628	7542450	ENSG00000255251	protein_coding	PRR23D1	proline rich 23 domain containing 1 [HGNC:49420]
8.1	7549053	7552062	ENSG00000248944	unprocessed_pseudogene	FAM90A6P	family with sequence similarity 90 member A6, pseudogene [HGNC:32254]
8.1	7556700	7559712	ENSG00000236660	unprocessed_pseudogene	FAM90A7P	family with sequence similarity 90 member A7, pseudogene [HGNC:32255]
8.1	7564350	7567360	ENSG00000234749	unprocessed_pseudogene	FAM90A21P	family with sequence similarity 90 member A21, pseudogene [HGNC:32269]
8.1	7571995	7575006	ENSG00000215365	unprocessed_pseudogene	FAM90A22P	family with sequence similarity 90 member A22, pseudogene [HGNC:32270]
8.1	7579644	7582653	ENSG00000241737	unprocessed_pseudogene	FAM90A23P	family with sequence similarity 90 member A23, pseudogene [HGNC:32271]
8.1	7592006	7592916	ENSG00000233176	unprocessed_pseudogene	OR7E157P	olfactory receptor family 7 subfamily E member 157 pseudogene [HGNC:31231]
8.1	7601004	7601267	ENSG00000254796	processed_pseudogene	RP11-1118M6.2	
8.1	7696830	7697093	ENSG00000254889	processed_pseudogene	AC084121.14	
8.1	7705182	7706113	ENSG00000254715	unprocessed_pseudogene	OR7E154P	olfactory receptor family 7 subfamily E member 154 pseudogene [HGNC:31309]
8.1	7715443	7718453	ENSG00000189393	unprocessed_pseudogene	FAM90A14P	family with sequence similarity 90 member A14, pseudogene [HGNC:32262]
8.1	7723091	7726101	ENSG00000231656	unprocessed_pseudogene	FAM90A18P	family with sequence similarity 90 member A18, pseudogene [HGNC:32266]
8.1	7730739	7733749	ENSG00000229477	unprocessed_pseudogene	FAM90A16P	family with sequence similarity 90 member A16, pseudogene [HGNC:32264]
8.1	7738387	7741396	ENSG00000224710	unprocessed_pseudogene	FAM90A8P	family with sequence similarity 90 member A8, pseudogene [HGNC:32256]
8.1	7746034	7749606	ENSG00000223535	unprocessed_pseudogene	FAM90A17P	family with sequence similarity 90 member A17, pseudogene [HGNC:32265]
8.1	7753682	7756692	ENSG00000237122	unprocessed_pseudogene	FAM90A19P	family with sequence similarity 90 member A19, pseudogene [HGNC:32267]
8.1	7761330	7764340	ENSG00000235825	unprocessed_pseudogene	FAM90A9P	family with sequence similarity 90 member A9, pseudogene [HGNC:32257]
8.1	7768977	7771988	ENSG00000254597	unprocessed_pseudogene	FAM90A10P	family with sequence similarity 90 member A10, pseudogene [HGNC:32258]
8.1	7778591	7781413	ENSG00000255378	protein_coding	PRR23D2	proline rich 23 domain containing 2 [HGNC:49396]
8.1	7793716	7795870	ENSG00000254776	unprocessed_pseudogene	AC084121.17	
8.1	7811720	7815716	ENSG00000186572	protein_coding	DEFB107A	defensin beta 107A [HGNC:18086]
8.1	7821966	7823889	ENSG00000186562	protein_coding	DEFB105A	defensin beta 105A [HGNC:18087]
8.1	7825172	7829053	ENSG00000186579	protein_coding	DEFB106A	defensin beta 106A [HGNC:18088]
8.1	7836471	7841242	ENSG00000176782	protein_coding	DEFB104A	defensin beta 104A [HGNC:18115]
8.1	7847876	7868867	ENSG00000178287	protein_coding	SPAG11A	sperm associated antigen 11A [HGNC:33342]
8.1	7881204	7882664	ENSG00000176797	protein_coding	DEFB103A	defensin beta 103A [HGNC:15967]
8.1	7891003	7892555	ENSG00000254543	processed_pseudogene	HSPD1P2	heat shock protein family D (Hsp60) member 1 pseudogene 2 [HGNC:5263]
8.1	7894629	7896711	ENSG00000171711	protein_coding	DEFB4A	defensin beta 4A [HGNC:2767]
8.1	7926337	7952413	ENSG00000215356	protein_coding	ZNF705B	zinc finger protein 705B [HGNC:32284]
8.1	7934412	7938770	ENSG00000229907	unprocessed_pseudogene	DEFB108P1	defensin beta 108 pseudogene 1 [HGNC:18089]
8.1	7955014	8008755	ENSG00000225725	lincRNA	FAM66E	family with sequence similarity 66 member E [HGNC:18735]
8.1	7967344	7968544	ENSG00000255211	unprocessed_pseudogene	RP11-1195F20.7	
8.1	7971661	7973253	ENSG00000237038	protein_coding	USP17L8	ubiquitin specific peptidase 17-like family member 8 [HGNC:37181]
8.1	7976393	7977985	ENSG00000225327	protein_coding	USP17L3	ubiquitin specific peptidase 17-like family member 3 [HGNC:37175]
8.1	7990415	7997521	ENSG00000205989	unprocessed_pseudogene	DEFB109	
8.1	8011782	8017011	ENSG00000233115	unprocessed_pseudogene	FAM90A11P	family with sequence similarity 90 member A11, pseudogene [HGNC:32259]
8.1	8019429	8024652	ENSG00000215354	ranscribed_unprocessed_pseudogen	FAM90A24P	family with sequence similarity 90 member A24, pseudogene [HGNC:32272]
8.1	8027077	8032305	ENSG00000254229	unprocessed_pseudogene	FAM90A12P	family with sequence similarity 90 member A12, pseudogene [HGNC:32260]
8.1	8039978	8040953	ENSG00000227013	unprocessed_pseudogene	OR7E96P	olfactory receptor family 7 subfamily E member 96 pseudogene [HGNC:14815]
8.1	8048919	8049267	ENSG00000253881	processed_pseudogene	RP11-52B19.8	

Chrom.linkloc	Gene start (bp)	Gene end	Gene ensembl ID	Gene biotype	Gene name	Gene description
8.1	8086022	8086765	ENSG00000255459	processed_pseudogene	RP11-52B19.10	
8.1	8088941	8089089	ENSG00000221305	miRNA	MIR548I3	microRNA 548i-3 [HGNC:35354]
8.1	8095946	8096726	ENSG00000244427	processed_pseudogene	RP11-52B19.1	
8.1	8116745	8116949	ENSG00000254311	processed_pseudogene	SNRPCP17	small nuclear ribonucleoprotein polypeptide C pseudogene 17 [HGNC:49832]
8.1	8154308	8216916	ENSG00000249188	unprocessed_pseudogene	ENPP7P1	ectonucleotide pyrophosphatase/phosphodiesterase 7 pseudogene 1 [HGNC:48699]
8.1	8167819	8226614	ENSG00000253893	antisense	FAM85B	family with sequence similarity 85 member B [HGNC:32160]
8.1	8188535	8189195	ENSG00000268955	lincRNA	RP11-556O5.6	
8.1	8228595	8244865	ENSG00000173295	ranscribed_unprocessed_pseudogen	FAM86B3P	family with sequence similarity 86 member B3, pseudogene [HGNC:44371]
8.1	8236003	8244667	ENSG00000253981	ranscribed_unprocessed_pseudogen	ALG1L13P	asparagine-linked glycosylation 1-like 13, pseudogene [HGNC:44382]
8.1	8317736	8386498	ENSG00000275342	protein_coding	PRAG1	PEAK1 related kinase activating pseudokinase 1 [HGNC:25438]
8.1	8414690	8424632	ENSG00000253505	lincRNA	CTA-398F10.1	
8.1	8456909	8461337	ENSG00000254153	lincRNA	CTA-398F10.2	
8.2	11982321	11984590	ENSG00000205883	protein_coding	DEFB135	defensin beta 135 [HGNC:32400]
8.2	11993174	11996312	ENSG00000205882	protein_coding	DEFB134	defensin beta 134 [HGNC:32399]
8.2	11999087	11999468	ENSG00000255174	processed_pseudogene	RP11-481A20.4	
8.2	12003400	12004082	ENSG00000254507	transcribed_processed_pseudogene	RP11-481A20.10	
8.2	12025244	12025598	ENSG00000255016	processed_pseudogene	RP11-481A20.8	
8.2	12033646	12034612	ENSG00000254817	unprocessed_pseudogene	OR7E160P	olfactory receptor family 7 subfamily E member 160 pseudogene [HGNC:31233]
8.2	12043506	12043670	ENSG00000254700	processed_pseudogene	RP11-1236K1.11	
8.2	12064389	12071747	ENSG00000233050	protein_coding	RP11-1236K1.1	
8.2	12087396	12087445	ENSG00000252029	rRNA	RNA5SP253	RNA, 5S ribosomal pseudogene 253 [HGNC:43153]
8.2	12095176	12099536	ENSG00000255544	unprocessed_pseudogene	DEFB108P3	defensin beta 108 pseudogene 3 [HGNC:26227]
8.2	12104389	12115516	ENSG00000215343	protein_coding	ZNF705D	zinc finger protein 705D [HGNC:33202]
8.2	12115782	12177550	ENSG00000255052	antisense	FAM66D	family with sequence similarity 66 member D [HGNC:24159]
8.2	12128107	12129298	ENSG00000254923	processed_pseudogene	RP11-1236K1.8	
8.2	12132417	12134438	ENSG00000226430	protein_coding	USP17L7	ubiquitin specific peptidase 17-like family member 7 [HGNC:37180]
8.2	12137168	12139077	ENSG00000223443	protein_coding	USP17L2	ubiquitin specific peptidase 17-like family member 2 [HGNC:34434]
8.2	12150895	12151134	ENSG00000254866	processed_pseudogene	DEFB109P3	defensin beta 109 pseudogene 3 [HGNC:30838]
8.2	12172761	12175771	ENSG00000205879	unprocessed_pseudogene	FAM90A2P	family with sequence similarity 90 member A2, pseudogene [HGNC:32250]
8.2	12178697	12182719	ENSG00000249889	unprocessed_pseudogene	ALG1L11P	asparagine-linked glycosylation 1-like 11, pseudogene [HGNC:44380]
8.2	12182096	12194133	ENSG00000186523	protein_coding	FAM86B1	family with sequence similarity 86 member B1 [HGNC:28268]
8.2	12194467	12196280	ENSG00000255495	antisense	AC145124.2	
8.2	12205759	12206389	ENSG00000254527	processed_pseudogene	ENPP7P12	ectonucleotide pyrophosphatase/phosphodiesterase 7 pseudogene 12 [HGNC:48697]
8.2	12290071	12296180	ENSG00000237215	unprocessed_pseudogene	ABC12-47043100G14.2	
8.2	12310962	12318316	ENSG00000232948	protein_coding	DEFB130	defensin beta 130 [HGNC:18107]
8.2	12333644	12333693	ENSG00000252535	rRNA	RNA5SP254	RNA, 5S ribosomal pseudogene 254 [HGNC:43154]
8.2	12341426	12345776	ENSG00000254623	unprocessed_pseudogene	DEFB108P4	defensin beta 108 pseudogene 4 [HGNC:30174]
8.2	12356136	12359391	ENSG00000215339	unprocessed_pseudogene	ZNF705CP	zinc finger protein 705C, pseudogene [HGNC:32283]
8.2	12362019	12388296	ENSG00000227888	lincRNA	FAM66A	family with sequence similarity 66 member A [HGNC:30444]
8.2	12374366	12375546	ENSG00000254423	processed_pseudogene	RP11-351I21.7	
8.2	12378679	12380265	ENSG00000255556	processed_pseudogene	RP11-351I21.6	
8.2	12393209	12400366	ENSG00000242296	unprocessed_pseudogene	DEFB109P1	defensin beta 109 pseudogene 1 [HGNC:18090]
8.2	12412827	12414373	ENSG00000270074	lincRNA	RP11-351I21.11	
8.2	12415080	12418090	ENSG00000251402	unprocessed_pseudogene	FAM90A25P	family with sequence similarity 90 member A25, pseudogene [HGNC:32246]
8.2	12421032	12425000	ENSG00000250794	unprocessed_pseudogene	ALG1L12P	asparagine-linked glycosylation 1-like 12, pseudogene [HGNC:44381]
8.2	12425614	12436343	ENSG00000145002	protein_coding	FAM86B2	family with sequence similarity 86 member B2 [HGNC:32222]
8.2	12448013	12511278	ENSG00000255549	unprocessed_pseudogene	ENPP7P6	ectonucleotide pyrophosphatase/phosphodiesterase 7 pseudogene 6 [HGNC:48698]
8.2	12476462	12477122	ENSG00000270154	lincRNA	RP11-419I17.2	
8.2	12537079	12665588	ENSG00000283674	lincRNA	RP11-419I17.1	
8.2	12570350	12571130	ENSG00000242607	processed_pseudogene	RPS3AP34	ribosomal protein S3a pseudogene 34 [HGNC:36293]
8.2	12580287	12581063	ENSG00000255122	processed_pseudogene	RP11-303G3.6	
8.2	12628476	12629256	ENSG00000244289	processed_pseudogene	RPS3AP35	ribosomal protein S3a pseudogene 35 [HGNC:35902]

Chrom.linkloc	Gene start (bp)	Gene end	Gene ensembl ID	Gene biotype	Gene name	Gene description
8.2	12638428	12638602	ENSG00000255253	processed_pseudogene	RP11-303G3.9	
8.2	12676035	12676389	ENSG00000254581	processed_pseudogene	RP11-303G3.10	
8.2	12684154	12685153	ENSG00000177400	unprocessed_pseudogene	OR7E8P	olfactory receptor family 7 subfamily E member 8 pseudogene [HGNC:8458]
8.2	12696307	12697273	ENSG00000254917	unprocessed_pseudogene	OR7E15P	olfactory receptor family 7 subfamily E member 15 pseudogene [HGNC:8386]
8.2	12703066	12704056	ENSG00000254724	unprocessed_pseudogene	OR7E10P	olfactory receptor family 7 subfamily E member 10 pseudogene [HGNC:8381]
8.2	12719132	12719190	ENSG00000264512	miRNA	MIR5692A2	microRNA 5692a-2 [HGNC:43466]
8.2	12721894	12756073	ENSG00000154359	protein_coding	LONRF1	LON peptidase N-terminal domain and ring finger 1 [HGNC:26302]
8.2	12727232	12727304	ENSG00000266206	miRNA	MIR3926-1	microRNA 3926-1 [HGNC:38959]
8.2	12727237	12727299	ENSG00000283523	miRNA	MIR3926-2	microRNA 3926-2 [HGNC:38883]
8.2	12765849	12811478	ENSG00000254813	lincRNA	RP11-252C15.1	
8.2	12794243	12818291	ENSG00000255494	lincRNA	LINC00681	long intergenic non-protein coding RNA 681 [HGNC:44423]
8.2	12945642	13031503	ENSG00000250305	protein_coding	KIAA1456	KIAA1456 [HGNC:26725]
8.2	12958387	12962200	ENSG00000251468	unprocessed_pseudogene	RP11-369K16.1	
8.3	87867601	87868607	ENSG00000273978	unprocessed_pseudogene	CTB-118P15.3	
8.3	87870743	87874068	ENSG00000176566	protein_coding	DCAF4L2	DDB1 and CUL4 associated factor 4 like 2 [HGNC:26657]
8.3	87974165	88019113	ENSG00000253171	antisense	CTB-118P15.2	
8.3	88032009	88328025	ENSG00000156103	protein_coding	MMP16	matrix metallopeptidase 16 [HGNC:7162]
8.3	88326836	88737134	ENSG00000253553	antisense	RP11-586K2.1	
8.3	88396437	88396547	ENSG00000251904	rRNA	RNA5SP272	RNA, 5S ribosomal pseudogene 272 [HGNC:43172]
8.3	88485110	88486094	ENSG00000250962	processed_pseudogene	RP11-69I13.1	
8.3	88808318	88808908	ENSG00000271156	lincRNA	RP11-642C5.1	

All gene biotypes are listed here. The exact intervals used to query Vega database were:

Chr2-1: 19417541-19746653

Chr2-2: 24680948-25585907

Chr5: 102620674-103702794

Chr7-1: 77653409-77985583

Chr7-2: 79635822-79894010

Chr8-1: 6986241 - 8316581

Chr8-2: 11840071-12880628

Chr8-3: 88564546-89894046
