# Supplementary material for: Microdeletion on chromosome 8p23.1 in a familial form of severe Buruli ulcer
Source: PLoS Negl Trop Dis. 2018 Apr 30;12(4):e0006429. doi: 10.1371/journal.pntd.0006429 (PMC5945055; doi:10.1371/journal.pntd.0006429)
Supplement: S1 List — List of participants according to their geographic location. (PDF) [file pntd.0006429.s004.pdf]

S1 List. Franco-Beninese Buruli Research Group. List of participants according to their affiliation.

The Franco-Beninese Buruli Research Group:

France:

Paris: Quentin B. Vincent[1,2], Jeremy Manry[1,2], Laurent Abel[1,2,4], Christian Johnson[3,5] , Alexandre Alcaïs[1,2]

Angers: Estelle Marion[6], Marie Kempf[7], Jane Cottin[7], Jean-Paul Saint-André[8], Laurent Marsollier[6]

Benin:

Pobè: Marie-Françoise Ardant[3,9], Ambroise Adeye[9], Aimé Goundote[9], Annick Chauty[3,9], Thierry Gateau[3,9]

Cotonou: Jean Gabin Houezo[10], Didier Agossadou[10]

(1) Laboratory of Human Genetics of Infectious Diseases, Necker Branch, Institut National de la Recherche Médicale (INSERM) UMR 1163, Paris, France

(2) Imagine Institute, Paris Descartes - Sorbonne Paris Cité University, Paris, France

(3) Fondation Raoul Follereau, Paris, France

(4) St Giles Laboratory of Human Genetics of Infectious Diseases, Rockefeller Branch, Rockefeller University, New York, NY, USA

(5) Centre Interfacultaire de Formation et de Recherche en Environnement pour le Développement Durable. Université d'Abomey-Calavi. Bénin

(6) INSERM UMR-U892 and CNRS U6299, team 7, University of Angers, CHU d'Angers, Angers, France

(7) Laboratoire de Bactériologie, CHU d'Angers, Angers, France

(8) Laboratoire d'Anatomie Pathologique, CHU d'Angers, Angers, France

(9) Centre de Dépistage et de Traitement de la lèpre et de l'Ulcère de Buruli (CDTLUB), Pobè, Benin

(10) Programme de Lutte Contre la Lèpre et l'Ulcère de Buruli, Ministère de la Santé, Cotonou, Bénin
